# Guidelines or mindlines? – implementing a new CKD guideline in German primary care

**DOI:** 10.1186/s12875-024-02589-w

**Published:** 2024-09-20

**Authors:** Konrad Laker, Tim Bothe, Natalie Ebert, Christoph Heintze, Elke Schaeffner, Karen Krüger

**Affiliations:** 1 Institute of General Practice and Family Medicine, Charité – Universitätsmedizin Berlin, Charitéplatz 1, 10117 Berlin, Germany; 2 Institute of Public Health, Charité – Universitätsmedizin Berlin, Berlin, Germany

**Keywords:** Guidelines, CKD, Quality improvement, Implementation, Mindlines

## Abstract

**Background:**

The development of clinical guidelines aimed at GPs is a key strategy to improving the management of chronic kidney disease (CKD). In 2019, the first CKD guideline aimed specifically at GPs practicing in Germany was published by the German College of General Practitioners and Family Physicians (DEGAM.)

**Aims:**

The aim of this study is to identify the barriers and enablers for the implementation of this guideline. The results of this project, together with quantitative evaluation against quality indicators for CKD in primary care will inform an update to the guideline.

**Methods:**

We performed 17 semi-structured interviews with GPs practicing in Berlin and Brandenburg. Transcripts were analysed using qualitative content analysis as described by Mayring.

**Results:**

We found that the perception of low clinical priority of CKD compared to other chronic diseases, opportunity cost of using guidelines, as well as poor patient understanding were significant barriers. GPs expressed that improved graphic design or integration of guideline recommendations in clinical decision support systems were enabling factors. Clinical problems concerning CKD were mostly solved by recourse to informal communication with specialists. GPs reported that they rarely consulted CKD guidelines as an aide to clinical decision making.

**Conclusion:**

The most significant barrier to use was that guidelines were not used as step-by-step decision aide in consultations with patients. Our analysis suggests that informal contact between primary and secondary care is significant conduit for evidence-based information on CKD in German primary care. Implementation projects should support the development of these relationships.

**Supplementary Information:**

The online version contains supplementary material available at 10.1186/s12875-024-02589-w.

## Introduction

Improving the detection, treatment and monitoring of non-dialysis dependent chronic kidney disease (CKD) in primary care has been the focus of quality improvement projects globally for more than 20 years [1]. CKD is an important independent risk factor for mortality [2], as well as for cerebro- and cardiovascular disease [3]. In Germany, 10% of the population are estimated to meet the criteria for CKD. The high prevalence of the disease means that CKD will be often initially detected and continuously managed by general practitioners (GPs.)

In Germany, the German College of General Practitioners and Family Physicians (DEGAM) – an academic, non-governmental organisation, supporting German GPs praticing evidence-based medicine – published the first German guideline on CKD aimed at GPs in 2019. Prior to its publication Germans GPs would have only had access to international guidelines on CKD, in particular recommendations issued by KDIGO (Kidney Disease: Improving Global Outcomes.) The new guideline was accompanied by a comprehensive two-page summary [4]. The guideline gives recommendations on investigations required for diagnosis, ideal monitoring intervals and treatments that minimise associated risks. Furthermore, guidance is given on which patients can safely be managed in primary care and those that may benefit from early assessement by nephrology. Referring patients at high risk of end stage kidney disease (ESKD) early is associated with lower mortality and better outcomes [5].

Guidelines for primary care in Germany are not routinely accompanied by a specific implementation strategy [6]. Furthermore, guideline adherence in primary care is not routinely monitored, sanctioned nor renumerated [7]. Therapeutic freedom - a doctor’s right to choose whichever treatment she considers clinically appropriate - is a central component of German doctors’ professional identity, meaning guideline recommendations are purely advisory and not legally binding in Germany. As the quality of primary care for CKD is not routinely measured, performance against quality indicators can only be estimated. One study of insurance claims data of a cohort of CKD patients treated in German primary care suggests that there is significant deviation from evidence-based recommendations by German GPs [4]: Only 20% of patients with an eGFR suggestive of CKD were given a coded diagnosis of CKD, indicating a low level of disease recognition or communication. Furthermore, the albumin creatinine ratio (ACR), a laboratory urine test crucial for the diagnosis of CKD and estimating the associated cardiovascular risk, is rarely ordered, suggesting that the diagnostic process considered the standard of care - and highlighted prominently in the guideline - is not followed in practice.

The large number of patients suffering from CKD and the small number of patients at risk of ESKD, mean that patients benefiting from nephrology review need to be carefully selected. Samples of insurance claims data indicate that inappropriate referral to nephrology (overtreatment), as well as delayed referral of patients with a high risk of progression (inadequate care provision) co-exist [8]. This suggests that only a small proportion of high-risk patients – those most likely to benefit from nephrology review – are currently referred early. Equally, there are many low-risk patients – suitable for management in primary care – which are inappropriately referred to nephrology.

The challenges and barriers to guideline-adherent treatment of CKD in countries where guideline adherence is central to the work of GPs due to funding being allocated to incentivise guideline-adherence, such as the Netherlands or the United Kingdom, are well established. A systematic review of 20 qualitative studies of GPs in North America, Australia, UK and the Netherlands showed that the most common barriers to CKD guideline use by GPs were lack of time, concerns that communicating the diagnosis of CKD might frighten patients, questions about the relevance of CKD to patients and dissatisfaction with existing CKD guidelines [9]. The most reported enablers for guideline-based treatment were the presence of supportive technology, such as clinical decision support systems (CDS), and a collaborative relationship with team members and nephrologists.

Given the large corpus of qualitative research answering our research question, we looked for a novel framework to assess the specific challenges and enabling factors that the implementation of the German CKD guideline might face. In 2011, Gabbay et al. [10] conducted ethnographic research in a specific British GP practice to understand the way GPs integrated evidence-based recommendations, such a guidelines, into their practice. They observed that guidelines were rarely used during consultations. The researchers were strong proponents of the evidence-based tools supporting clinicians and attempted to understand why many recommendations were not followed in practice.

Their explanation for the barriers to guideline adherence by GPs was that guidelines focussed on only the clinical side of decision making. Doctors were found to balance a variety of factors, such as financial costs, relative health benefits and managerial targets, in their clinical practice [11]. The term “mindlines” attempts to capture the complex psychological factors bearing on the clinical decision making process of GPs during a consultation. Mindlines are defined as the tacit internalised guidelines GPs initially developed in their early training and subsequently adapted mainly in response to discussion within the team, external opinion leaders, and brief readings. Mindlines unlike guidelines are context-specific and developed in response to informal reading, discussion with colleagues and negotiation with patients. The idea of mindlines explains why clinicians open to evidence-based practice may be slow to integrate guidelines recommendation into their clinical practice. In contrast to approaches focussing on barriers and enabling factors faced by individual doctors adopting evidence-based practices, mindlines are created and refined in social networks of colleagues.

### Purpose or research question

This study was commissioned to identify the specific challenges and enabling factors for the implementation of the first DEGAM CKD guideline for GPs practicing in Germany. Anticipating an overlap with existing literature, we wanted to assess whether a mindline framework would help us elucidate the possible barriers in accepting the guideline and whether it was still suitable nearly two decades after similar guidelines had been introduced in other countries.

This study is part of the larger GUIDAGE-CKD project, which aims to analyse the quality of CKD care in German primary care after the introduction the DEGAM CKD guideline. As part of the larger study project, quality indicators for CKD have been developed and will be evaluated against insurance claims data sets. Results of this project have supported an update to the guideline.

## Methods

### Qualitative approach and research paradigm

We conducted 17 semi-structured interviews between October 2022 and March 2023 with GPs practicing in Berlin and Brandenburg until we reached theoretical saturation. Two pilot interviews were used to assess the validity of the interview guide initially formulated by the authors and based on literature and expert opinions. The interview guide was subsequently reviewed in internal research workshops and a final draft agreed upon with the larger GUIDAGE-CKD research group. The semi-structured interviews were conducted by the lead author, a male GP registrar, face-to-face at the GP practices and lasted 35–65 min. No other participants were present during the interviews. In preparation, the lead author received formal training on how to conduct qualitative interviews. No repeat interviews were conducted. The interview guide is provided in the supplementary files. During the last third of the interview, a copy of the 2019 summary of the DEGAM CKD guideline was shown to the participating GPs and evaluated qualitatively.

### Sampling strategy

GPs practicing in Berlin and Brandenburg (either salaried GPs or GP partners) were recruited through the RESPoNsE research network based at the Institute of General Practice and Family Medicine, Charité – Universitätsmedizin Berlin. 312 GPs were invited by email through the network. We also recruited from other independent GP practices to ensure that the sample represented a maximum variation of diverse characteristics such as sex, practice type, size, and grade of urbanisation, including the socioeconomic profile of the patients. For each practice, only one participant was selected. 17 fully qualified GPs from 17 different practices were recruited to participate in this study.

### Human ethics and consent to participate declaration

All participants provided written informed consent prior to participation in this study. The lead author informed the participants about his work as a GP registrar, interest in publishing the results with a view to gain a MD, and funding arrangements. The study protocol was approved by Charité Ethics Committee (EA1/050/22), Charité – Universitätsmedizin Berlin, Germany and all research was conducted in accordance with the principles of the Declaration of Helsinki.

### Data collection methods

In addition to the interview recordings, we documented the demographic details of all participants, as well as information on time since graduation, specialist qualification and information on participation in continuing professional education, and membership in professional organisations. This data was collected using a questionnaire completed by the participants at the time of the interview.

### Data collection instruments and technologies

Interviews were digitally recorded using a Grundig Digta 7 voice recorder and transcribed verbatim by the external transcription company TranskriptWunder. Transcripts were not returned to participants for comment or correction.

### Data processing

A comprehensive data protection plan was agreed with the Charité Data Protection Officer (CTO-Nr: 688, ePA: 20017266). Digital files of audio recording, transcripts and analysis codes will be stored securely on Charité servers for 10 years.

### Data analysis

We used the software MAXQDA 2022 to perform qualitative data analysis as described by Mayring, a form of systematic, rule-guided, qualitative text analysis. Two procedures are central: the inductive category development and the deductive category application approach to identify, analyse, and report patterns in the data [12]. We utilised a mixture of deductive codes and inductive codes developed from the material. Intercoder variability was reduced by utilising a second reviewer. Different aspects were discussed and revisions were made until a common approach was agreed. The code system was presented and discussed once again in an internal research workshop. In presenting the data, we choose to maintain the categories of barriers and enabling factors set in our research question but tried to highlight the limitations of this in our discussion. Thoughtfully selected quotes were used to explicate the subjects. We did not seek participants feedback on the findings.

## Results

**Table 1:** Demographics of Study Population

Mean age (SD)	46.9 years (10.5)
Number of female GPs (%)	35.3%
Mean years since graduating from medical school (SD)	19.5 years (11.1)
Mean years since GP specialist qualification (SD)	13.2 years (10.7)
Number of practices in each location (%)	
Berlin	8 (52.9%)
Brandenburg	7 (47.1%)
Number of GPs with membership for professional society (DEGAM) (%)	12 (70.6%)
Mean number of fully qualified GPs per practice (SD)	1.9 doctors (1)
Mean number of GP trainees per practice (SD)	0.75 trainees (1)
Mean number of registered patients per practice (SD)	1525 patients (778.2)
Number of participants engaged in teaching medical students (%)	12 (70.6%) of participants taught medical students
Number of participants who were members of quality improvement groups (Qualitätszirkel) (%)	13 (76.5%) of participants were members of Qualitätszirkel

### Sample representativeness

GPs interviewed in our study were younger with an average age of 46,9 years compared to the German average GP in 2023 of 55,2 years [13]. GPs in our sample were more likely to be male 64,7% compared to the German average in 2023 of 49,5% [14]. No high quality comparative data exists for the other parameters (Table 1).

### Observations on guideline use in general

Most GPs had never consulted the DEGAM guideline on CKD prior to the interview or had done this for the first time in preparation for the interview. In general, all GPs felt confident about how to find and access DEGAM guidelines. At the time of writing, thirty guidelines had been published on the DEGAM website for a variety of presenting complaints and diseases. DEGAM was described as a trustworthy and authoritative source of evidence-based information. Access to guidelines was not described as a barrier to guideline use.

*I only have to double click to access the guideline (…). It’s really quick (…) and I no longer have to print anything.***HA03** Berlin

*[…] I must admit that I don’t check who the authors are. I trust DEGAM (laughs) to do good work and that they have selected good people to produce this guideline.***HA08** Berlin

Guidelines were thought to outline a general approach to a disease most useful during preparation for qualifying exams. One GP described consulting guidelines for the first time in preparation for exams with their practical use declining afterwards.

*For someone new to general practice*, *this is a great*, *GREAT way to approach a problem. However*, *for someone like me*, *who has been practicing for several years*, *(…)*, *less so. I would probably not consult this [guideline].****HA13****Brandenburg*

Rather than being seen as step-by-step recommendations to be used in daily practice, guidelines were understood as sources of legitimatisation in the case of litigation or requests by health insurance providers to justify treatment decisions.

*What’s interesting is that I do not consult the guideline to be guided by it*, *but rather that I read the guideline to feel validated or challenged in my decisions.****HA14****Brandenburg*

### Barriers specific to CKD guideline recommendations

GPs commonly described CKD as a disease of low clinical priority. GPs only rarely observed patients progressing from CKD to ESKD requiring renal replacement therapy. GPs appreciated that their CKD patients had an elevated cardiovascular risk, but often attributed to the associated co-morbidities, rather than CKD independently. Concerns about kidney function or CKD are rarely the primary reasons for patients seeking a consultation. A significant barrier to communicating the diagnosis was the perception that patients did not understand CKD or its associated risks well.

*The way the kidney functions is not a priority for the patient. They do not see or fail to understand what happens if the kidney is not working well. And if I try and explain this*, *it is rarely relevant to patients.****HA03****Berlin*

*If I had to compare this with cardiovascular things or Diabetes. For me this is somewhat connected. However*, *well*, *I don’t see this [CKD] as a separate*, *isolated unit.****HA05****Berlin*

*[…]I have been running this practice for six years. I think*, *I have maybe had two patients who have required dialysis.****HA06****Brandenburg*

Some GPs felt that an understanding of CKD was rarely helpful to patients as patients had little control over the disease. Most GPs acknowledged the importance of patients being aware of their kidney function, especially when buying over-the-counter medications such as nephrotoxic Ibuprofen. GPs were aware that the term ‘kidney failure’ may frighten patients, but the majority did not consider this a significant barrier to communicating the diagnosis. The most significant barrier to effective communication was a perceived lack of relevance to the patient and the lack of individual therapeutic options.

*[…] meanwhile for chronic kidney disease – admittedly purely subjectively – I feel*, *all I can do is observe and make SLIGHT*, *well*, *minute adjustments.****HA01****Brandenburg*

Guidelines in general were seen as less useful for older patients with complex needs and multiple chronic conditions, the most common demographic to be at risk of CKD.

*And this guideline – in my opinion – is a higher-level structure that describes a typical process. I*, *however*, *need to think about this problem differently for each patient. Age*, *co-morbidities*, *quality of life*, *life expectancy.****HA14****Brandenburg*

The most cited reason for not consulting guidelines in clinical practice was lack of time. More precisely, many GPs highlighted the opportunity cost of using guidelines during a consultation. Several GPs chose to refer patients due to time constraints rather than read up on guideline recommendations. GPs also described spending a significant amount of time on informal exchanges with nephrologists, e.g. via fax or trying repeatedly to reach a specialist colleague by phone. Guideline use was described to be in direct competition with the limited time available for patient care, mirroring recent concerns about the time needed to treat for guideline adherence [15].

*[…] If guidelines give very general recommendations*, *the trade-off between the time required to read a guideline and its benefit to my clinical practice is not favourable*, *given my daily schedule. Time is always short. While I am reading guidelines – which may take several hours – I do not have time to*, *for example*, *care for patients.****HA01****Brandenburg*

*There are simply too many people and too little time*, *obviously it’s a luxury to do everything by the book and to a high standard etc. If somebody comes to me*, *I have ten minutes*, *more or less. And I have to discuss a million things and then I also have to counsel them about chronic kidney disease. I have to set priorities*, *what is important right now and for the patient in front of me.****HA05****Berlin*

Many GPs described poor graphic design, especially a strong focus on purely written text and lengthy chapters, as a significant barrier to everyday use. Some GPs expressed a strong preference for having all relevant information accessible at a glance to optimise usability within the confines of a short consultation. The density of print, the absence of visual algorithms, and the lack of key messages for GPs were criticisms specific to the summary of the DEGAM CKD guideline jointly evaluated during the interview.

*Yes*, *I think*, *lack of visual clarity is a barrier.****HA01****Brandenburg*

*MAYBE*, *more graphic design*, *diagrams would/might be helpful. A LITTLE less text*, *more flow charts.****HA10****Berlin*

### Enabling factors for using the CKD guideline

The most significant enabling factor for guideline use was considered informal communication with nephrologists, practice partners, junior doctors, and medical students. Informal exchange with nephrologists via telephone or instant messaging was considered crucial for deciding which patients to refer and for receiving advice on how to adjust the complex medication regimes of older co-morbid patients. Many GPs felt that they were kept up to date on the most recent evidence and guidelines through exchange with their specialist colleagues.

*Well*, *I do NOT look at guidelines (laughs) in the moment. I trust the knowledge*, *I have accumulated previously or acquired through further training. Probably*, *the most likely route I would take*, *would be to seek a discussion with colleagues*, *such as the nephrologist, and ask them how to proceed.****HA01****Brandenburg*

*Well*, *I usually try - if there are changes to guidelines or maybe paradigm shift within a particular guideline - to discuss these with my colleagues*, *get their take on it before I adopt something.****HA10****Berlin*

GPs described selecting nephrologists to refer to based on their availability for exchange. A significant hurdle in standardising referral criteria was the perception by GPs that individual nephrologists often had divergent criteria on when patients would benefit from review. GPs based in Berlin – in contrast to those in Brandenburg – were often unclear about the division of labour between nephrology and primary care. Many GPs appreciated patients being reviewed by nephrologists and discharged back to their care after review with a specific plan.

*It varies*, *I have to say*, *I have had very DIFFERENT experiences. It depends extremely on the practice. However*, *I have also had positive experiences*, *when I received very CLEAR instructions and follow up appointments.****HA08 Berlin***

*If I am undecided*, *I pick up the phone and try to contact a colleague.****HA16 Berlin***

Teaching of both GP registrars and medical students was considered important to stay up to date with guidelines. The possibility to exchange with colleagues in *Qualitätszirkel*, formal meetings with colleagues to develop quality improvement projects, was considered an important method to evaluate guidelines and integrate them into clinical practice.

*And we have a quality improvement group in our practice*, *(…) we discuss cases or conditions in an evidence-based manner (…) evidence plays an essential role in these meetings and we also present guidelines*, *yes.****HA09****Berlin*

*The students placed here*, *which we train*, *they often bring a breath of fresh air. And through them*, *I also often receive new information*, *which I would otherwise NOT been made aware of.****HA13****Brandenburg*

GPs reported that they were not supported by their electronic patient record (EPR) to be more guideline adherent. Clinical decision support systems for key recommendations or automated case finding for CKD were understood to be theoretically helpful as they are currently not available in Germany.

*Well*, *this would be/this automatic support is not bad. That’s missing here COMPLETELTY. Who knows how often I forget something.****HA02****Berlin*

*[…] well*, *we really should perform an internal audit and know our EPR*, *that could run through our data to answer this type of question. Who has CKD and how many are – whatever – taking Ibu[profen] and so forth […]’****HA04****Berlin*

## Discussion

### Summary

Our findings show significant overlap with the recent systematic review of challenges and enabling factors for the implementation of CKD guidelines. Time constraints were also the most cited reason for guideline non-adherence in our study. GPs were less concerned about frightening their patients about the diagnosis. However, GPs also expressed that communication about the disease due to poor patient understanding of kidney function was a barrier. GPs felt that the DEGAM guideline usability could be improved through better graphic design as well as incorporating flow charts and key messages, which matches the dissatisfaction GPs described with current guidelines in the systematic review. GPs also reported a good collaborative relationship within the team and with nephrologists as a significant enabler to guideline use. GPs were open to supportive technology but had little access to this. Our current study suggests that the barriers and enablers for guideline change in Germany are very similar to those described in other countries.

### Strength and limitations

Participating GPs were recruited via a research network and were all motivated to practice evidence-based medicine, which may not be representative of German GPs. Our sample of GPs was more likely to be younger and male than the average German GP. This limitation is also a strength as it highlights the concerns of younger GPs most likely to engage with guidelines. Only GPs practicing in Berlin and Brandenburg were included in the study, which may limit the transferability of findings to other parts of Germany and internationally. A limitation of conducting qualitative interviews is that we have only captured how GPs described their own thinking or actions. An ethnographic research design may have also allowed us to analyse how GPs utilise evidence in practice.

### Comparison with existing literature

At the beginning of our research, we understood the implementation of the guideline as a technical problem, requiring the identification of enabling factors and elimination of limiting factors. The analysis of our interviews yielded a more complex picture. This group of German GPs – particulary open to evidence-based recommendation – did not use guidelines as intended by the guideline authors.

Gabbay et al. incidentally observed the introduction of the 2006 CKD guideline in the UK during their ethnographic research [10]. Like their German colleagues in 2022, they questioned the relevance of the CKD diagnosis to their patients and their own decision making. Guideline recommendations were helpful not as a step-by-step guide but used to refine their own internalised mindlines by discussing them in practice meeting or appointing a local CKD lead. Guideline recommendations were only accepted after further discussion with colleagues and other opinion leaders. In summary, analysing our interviews considering the mindline theory finds similar patterns in how GPs treat guidelines today compared to those nearly two decades ago by Gabbay et al.

### Implications for research and practice

The most significant barrier to guideline use is that GPs do not use guidelines in clinical practice in the way they were designed, which is with an intention to guide actual step-by-step decision making. Nevertheless, this also highlights which factors might enable guidelines to be more relevant in everyday practice. Interaction with colleagues, e.g., discussing new guidelines in practice meetings or asking medical students to present new guideline material, are exactly the mechanisms that, in the framework of mindlines , lead to changes in clinical practice. Similarly, informal contact with nephrologists is quick, cheap and often preferred over standardised and slow written communication. Many GPs spend most of their training in hospitals where this form of information exchange is practiced. Currently, this crucial cost-effective method of ensuring that CKD patients are cared for in the right setting is not reimbursable by German GPs or nephrologists. Recognising that both primary and secondary care will benefit from a collaborative working relationship is crucial for the guideline’s recommendations to be adopted.

While these points apply to guideline implementation projects more generally, we also found recommendation specific to CKD. If guidelines are not consulted regularly, key messages – such as the need to measure ACR before making the diagnosis of CKD – could be implemented in the form of clinical decision support systems inside the electronic patient record. Similarly, integrating information on renal dosing of drugs in the presence of reduced eGFR could be integrated into prescribing software. Overall the large heterogeneity of different EPR systems in use in Germany is a practical hurdle [16], however, the GPs in this study were open to be supported by these systems.

Gabbay et al. developed their theory in response to an increasingly rigid quality improvement architecture surrounding General Practice in the UK, which did not consider the complexity of clinical decision making. The relatively late introduction of a CKD guideline aimed at GPs represents a significant opportunity. German GPs enjoy a larger degree of professional autonomy [17], which may allow them to reframe the debate around guideline implementation for CKD utilising the accumulated evidence of their international peers.

## Conclusions

Guidelines for CKD are rarely consulted by the GPs interviewed in this study. Adopting a more complex theory on how clinical decisions are made by doctors, such as mindlines *m*, could help us to reframe the problem of guideline adherence. Rather than imposing complex and expensive implementation strategies which may themselves be time-consuming while producing limited clinical benefit to patients, the point of view of the end-user should be a central concern for guideline authors.

CKD is a difficult condition to design a guideline for as it is a largely asymptomatic disease poorly understood by patients. As a result, it is not a common reason to seek medical advice and GPs do not understand it to be an isolated disease, but often seen as a co-morbidity particularly of their complex geriatric patients. Despite the link to all-cause mortality and cardiovascular risk, GPs may underestimate the role of CKD due to the low rate of ESKD. When complex clinical problems arise in this patient group GPs often consult nephrologists informally to guide their decision making, rather than guidelines.

Mindlines are collectively constructed, meaning that changes to clinical practice arise from discussion with other trusted clinicians in addition to evidence-based information from guidelines. Quality improvement projects targeting the quality of care of CKD may want to focus on improving the collaborative relationship between GPs and nephrologists.

## Electronic supplementary material

Below is the link to the electronic supplementary material.

Supplementary Material 1

## Data Availability

The interview guide is provided in the supplementary files. The data that support the findings of this study are available on request from the corresponding author, K.L. The data are not publicly available due to their containing information that could compromise the privacy of research participants.

## Abbreviations

ACR: Albumin Creatine Ratio
CDS: Clinical Decision Support System
CKD: Chronic Kidney Disease
DEGAM: Deutsche Gesellschaft für Allgemeinmedizin und Familienmedizin
GFR: Glomerular Filtration Rate
ESKD: End Stage Kidney Disease
KDIGO: Kidney Disease: Improving Global Outcomes
QOF: Quality Outcomes Framework
RRT: Renal Replacement Therapy
