# Supplementary material for: Guidelines or mindlines? – implementing a new CKD guideline in German primary care
Source: BMC Prim Care. 2024 Sep 20;25:344. doi: 10.1186/s12875-024-02589-w (PMC11414130; doi:10.1186/s12875-024-02589-w)
Supplement: Supplementary file 1 — Supplementary Material 1 [file 12875_2024_2589_MOESM1_ESM.docx]

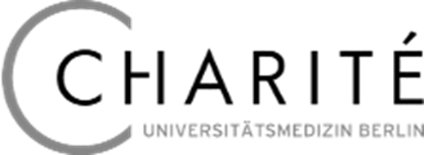
Interview Guide – GUIDAGE-CKD

*Dr Karen Krüger and Konrad Laker, Institute of General Practice and Family Medicine, Charité Berlin*

## Research Question

Which **challenges** to GPs experience in implementing the S3-gudieline für non-dialysis dependent chronic kidney disease?

Which **solutions** are effective for the implementation of this guideline?

## Introduction

Thank you for agreeing to participate in this study! I would like to provide you with some background information on our research. This study is part of a larger research project. Our research group specifically wants to understand how healthcare of older adults with CKD is provided in general practice. We know that chronic kidney disease is an important risk factor for cardio- and cerebrovascular disease. It is also associated with increased mortality. Despite this, chronic kidney disease does not receive the same recognition as other chronic disease, such as hypertension or Diabetes.

The aim of today’s interview is not to test your knowledge about CKD or to check whether you are following guideline recommendations. We are interested in your opinion as an expert in dealing with CKD patients on a daily basis. We want to understand which challenges and enabling factors you see for the evidence-based clinical care of this particular group of patients.

The recording of today’s interview will be transcribed by an external agency and subsequently evaluated by my colleagues. Your experiences and opinions are valuable to us, as this research will inform an update to the guideline in 2024. It may also guide us in our efforts to implement the guideline. At the end of the interview, I would like you to complete a short questionnaire which will help us put the interviews into context.

## Opening Question

Can you tell me about a **clinical case** where CKD played an important role for you?

## Interview Guide

| Themes | Narrative questions | Guiding questions | Background information |
| --- | --- | --- | --- |
| **Patient factors**  Perception, acceptance, behaviour | In which **clinical situation** do you normally make a diagnosis of CKD?  Do patients **present** with concerns about CKD? | 1. Does the treatment of CKD stand in conflict with the treatment of other **co-morbidities**?  2. Does age play a role in your perception of **guideline-adherent treatment**?  3. Does age play a role for the **communication** of a CKD diagnosis?  4. Which clinical benefit do see in **communicating** the diagnosis of CKD to an older patient? | **Incidental finding.** Have patients asked you about CKD after having been alerted to this by lab sheets or hospital discharge letters? How to you deal with these requests? |
| **Behaviour**  Implementation and actions, availability and usability | In which clinical situation does CKD play an important role for you? | 1. How does the presence of CKD influence your **clinical decision making**?  2. What role does CKD play when prescribing **medications**?  3. Which are crucial factors in your decision about how to **refer** to a nephrologist?  4. Which **guideline formats** are the most appropriate for your use, i.e. summaries or cards | **Potential** roles for CKD  - Dosing of medications  - Elimination of nephrotoxic medications  **Access** routes to guidelines  - Print publications  - Online via non-profit or commercial solution  - Discussion in quality improvement groups or professional congresses  Reasons for referral  - Urine dipstick vs laboratory ACR  - Rapidly decreasing eGFR  - Age of the patient |
| **Acceptance**  Opinion of the guideline and motivation | Which **information** **resources** do you use in your every-day work with CKD patients? | 1. What are your **opportunities** for guideline use? What **triggers** you to read a guideline?  2. Which **factors** are important for your **assessment** of guideline quality?  3. What do you think might be **barriers** in your use of this guideline?  4. Did you use guidelines more frequently during **university** of your **post-graduate training**? | Factors impacting assessment of **guideline** **quality**  - Own assessment of existing evidence base  - Reputation of journal or issuing organization  - Recommendations from colleagues or interaction with specialists  Potential barriers  - Limited impact on clinical decision making of CKD  - Lacking financial incentives for guideline use |
| **Solutions**  *Known and proven ways of implementing guidelines*  *Discussion of known and effective implementation strategies* | What would help you to use the guideline in your everyday practice? | 1. Have you had **positive experiences** with **guidelines** and how have they changed your clinical practice?  2. Are you using **quality improvement methods** and have you been able to integrate them into your practice?  3. Have you experienced specific guideline **implementation strategies?**  4. Are you performing regular **audits** to measure your performance? | Evidence-based **implementation strategies**  - External audits and comparison with colleagues, e.g. in quality improvement groups  - Computer-based clinical decision support systems or focused reminder systems  - Practice-based teaching and training |
| **Perception**  How is the guideline received  Knowledge of guideline contents | The short summary of the DEGAM guideline on CKD will be evaluated together with the GPs. What is your **initial impression** of the guideline? | 1. Which guideline recommendations would **change** your existing clinical practice?  2. Do the recommendations match your **clinical practice**? Are there **differences**?  3. Are **crucial** **take-home points** easy to identify?  4. How would you rate the **graphic design**? |  |
| **Ideas for implementation and expectations** | Do you have specific ideas for the **presentation** or **use** of the guideline? | Are guidelines the right **tool** for providing you evidence-based information on CKD? |  |
